# Economic Impacts of Initiating Vaccination at 3 Months vs. 6 Months in an Influenza Pandemic in the United States

**DOI:** 10.3390/vaccines13080828

**Published:** 2025-08-01

**Authors:** Van Hung Nguyen, Pascal Crepey, B. Adam Williams, Verna L. Welch, Jean Marie Pivette, Charles H. Jones, Jane M. True

**Affiliations:** 1 VHN Consulting Inc., Montréal, QC H2V3L8, Canada; 2 Ecole des Hautes Etudes en Sante Publique, 35043 Rennes, France; 3 Pfizer, 66 Hudson Boulevard East, New York, NY 10001, USA; adam.williams@pfizer.com (B.A.W.);

**Keywords:** influenza, pandemic, vaccine, cost, speed

## Abstract

**Background/Objectives:** An influenza pandemic is likely to occur in the coming decades and will be associated with substantial healthcare and financial burdens. In this study, we evaluated the potential economic costs of different vaccination scenarios for the US population in the context of a moderate or severe influenza pandemic. **Methods:** Economic analysis was performed for initiation of pandemic vaccination from 3 months vs. 6 months in the US after declaration of a pandemic. We evaluated three vaccine effectiveness levels (high, moderate, low) and two pandemic severity levels (moderate and severe). **Results:** No vaccination would lead to total direct and indirect costs of $116 bn in a moderate pandemic and $823 bn in a severe pandemic. Initiation of vaccination at 3 months would result in cost savings versus no vaccination (excluding vaccine price) of $30–84 bn and $260–709 bn in a moderate and severe pandemic, respectively, whereas initiation of vaccination at 6 months would result in cost savings of $4–11 bn and $36–97 bn, respectively. Cost savings of $20 bn and $162 bn would occur in a moderate or severe pandemic, respectively, from use of a low effectiveness vaccine from 3 months instead of a high effectiveness vaccine from 6 months. **Conclusions:** Rapid initiation of vaccination would have a greater impact than increased vaccine effectiveness in reducing the economic impacts of an influenza pandemic.

## 1. Introduction

Given the risk of antigenic shift or crossover from animal reservoirs, a future influenza pandemic is highly likely to occur in the coming decades. In the aftermath of the COVID-19 pandemic, effective preparedness efforts are needed to minimize the disease and economic burden of any future pandemics on societies. The COVID-19 pandemic had substantial health and economic costs: in addition to the ~7 million deaths globally and substantial direct (e.g., morbidity and long-term symptoms) and indirect (e.g., delayed cancer care, increased mental health disorder diagnoses) health impacts of COVID-19, the pandemic also had a huge financial effect, resulting in a global drop in Gross Domestic Product (GDP) of 3.4% [1]. Excluding the direct healthcare costs of vaccination and medical care of acute symptoms, the economic impacts of the COVID-19 pandemic in the United States (US) have been estimated at $16 trillion, including lost GDP, loss of income from the pandemic-induced recession and lockdown measures, and economic effects of long-term physical and mental health impacts [2].

Vaccination plays a critical role in mitigating the transmission of novel viruses and viral strains among populations that are predominantly immunologically naïve to the new threat. While vaccines to currently circulating influenza strains are used annually in national vaccination programs, development of vaccines specifically targeting newly emerged influenza strains could be expected to take at least six months, based on the experience of the 2009 pandemic [3]. While technological advances during the COVID-19 pandemic may shorten these timelines, it is still likely there would be a lag of several months between identification of a pandemic strain and roll-out of mass vaccination due to regulatory, manufacturing, and logistical constraints [4,5].

As part of preparedness efforts for future pandemics, the US government initiated the US National Pre-Pandemic Influenza Vaccine Stockpile (NPIVS) program which maintains a library of influenza virus vaccine seeds of strains considered to be of pandemic potential [6]. The goal of this initiative is to reduce the time to produce and deliver effective vaccines during a pandemic, as well as potentially allowing dose sparing to increase availability of individual doses. These vaccines are likely to therefore be available in a shorter timeframe than vaccines which are developed after emergence of the new pandemic strain, but may be less effective than specifically designed vaccines. In a previous analysis, we noted that the speed of initiation of a vaccine campaign is key to minimizing the health impacts of pandemic influenza in the US, with roll-out of vaccination to the most vulnerable groups from 3 months after declaration of a pandemic leading to substantial in reductions in symptomatic cases (23–78%), outpatient cases (26–81%), hospitalizations (28–85%), and deaths (32–94%) compared with vaccination from 6 months. Additionally, use of a low effectiveness vaccine from 3 months was found to be more impactful in reducing negative health outcomes than a highly effective vaccine from 6 months [7].

Complementary to the previous analysis on the epidemiological impacts of early versus late vaccination in an influenza pandemic in the US, this analysis evaluates the economic impacts of the speed of initiation of mass vaccination across multiple vaccine effectiveness and pandemic severity scenarios in terms of direct and indirect costs at a population level, and for individual age groups.

## 2. Materials and Methods

### 2.1. Study Objective

The primary objective of the current analysis was to evaluate the economic impacts of initiating influenza pandemic vaccination from 3 months after pandemic declaration versus 6 months after pandemic declaration in the US. Evaluation for costs per individual age group was performed as an additional exploratory objective.

### 2.2. Epidemiological Model of Influenza Transmission and Health Outcomes

As part of this analysis, a previously described epidemiological model [7] was used to model dynamics of a hypothetical influenza pandemic (Table S1) [8,9,10,11,12,13,14,15,16,17,18,19,20,21,22,23]. In brief, we used an age-structured susceptible, exposed, infected, recovered (SEIR) model to evaluate influenza transmission and vaccination impacts during a pandemic situation, combined with a static model which translated infection rates into health outcomes and economic burdens for patients at lower- or high-risk of severe influenza outcomes.

As part of the analysis, three vaccine effectiveness scenarios were assessed. The high effectiveness scenario assumed an effectiveness of 92% against symptomatic disease and 97% against hospitalization, based on data from mRNA COVID-19 vaccines [8,9,12]. The moderate effectiveness scenario assumed varying effectiveness by age group (46–88% against symptomatic disease and 64–88% against hospitalization), based on data from 2009 pandemic A/H1N1 influenza vaccines [10]. The low effectiveness scenario was based on seasonal estimates of vaccine effectiveness against A/H3N2, with vaccine effectiveness varying across age groups from 24% to 43% against both symptomatic and hospitalized influenza [11]. A no vaccination scenario was included in the analysis to evaluate the absolute costs of vaccination. All scenarios assumed the use of antivirals for 25% of patients with symptomatic infection, based on stockpiling recommendations [19], with effectiveness varying with age between 18% and 70% [18]. Vaccine coverage was based on data from the COVID-19 pandemic combined with seasonal influenza coverage rates in the US (Table S2) [13,14].

Assumptions of probabilities of health outcomes were based on data from previous moderate and severe influenza pandemics [20] and calibrated to the US contact matrix used in this model, using a maximum likelihood approach. Mortality was projected to mainly affect infants and older adults, and was modelled as a J curve. Further details of epidemiological model assumptions are provided in Table S1. As outline in [7], the pandemic virus was assumed to emerge during the Southern hemisphere influenza season, with a pandemic being declared in mid-summer. US cases were assumed to follow the same pattern as seasonal influenza, emerging in the autumn and peaking in late winter.

### 2.3. Economic Assumptions

Probabilities of outpatient and hospital visits, and associated costs varied by age group (Table S3) [24,25]. Antiviral treatment and vaccine administration were assumed to cost $50 each per person. As no details were available for vaccine prices, these were not included in the cost calculations (see discussion). Indirect costs were calculated using a human capital model, based on the disease impact on loss revenue due to absenteeism, and loss of future earnings due to mortality [26].

### 2.4. Vaccination Scenarios

Two vaccination scenarios were evaluated. In the first scenario, vaccination of the high-risk population was assumed to begin at 12 weeks (3 months), ramping up to full coverage over the following 12 weeks (61% coverage of modelled coverage level across age groups after 4 weeks of vaccination). Vaccination of the low-risk population started at 24 weeks after declaration of the pandemic, with a similar ramp up function. The second scenario assumed vaccination of the high-risk population from 24 weeks (6 months) and of the low-risk population from 36 weeks, using the same exponential ramp up function. The high-risk population included individuals with ≥1 medical condition putting them at increased risk of influenza complications together with all individuals ≥65 years of age. All other individuals ≥6 months of age were considered to be low-risk. Individuals <6 months of age were not considered in the model as they are not currently eligible for vaccination against influenza in the US.

Both vaccination scenarios were evaluated for a combination of pandemic profile (moderate or severe) and vaccine effectiveness (low, moderate, high). Estimates of pandemic severity were based on data from Monto et al. [20], with higher rates of hospitalization, outpatient visits, and deaths in a severe pandemic (see Table S1).

### 2.5. Subgroup Analysis

As a subgroup analysis, costs were evaluated independently for each of the age groups included in the epidemiological model (6 months to 4 years, 5 to 17 years, 18 to 49 years, 50 to 64 years, 65 to 74 years, and ≥75 years) for each of the vaccine effectiveness, severity and timing of vaccination initiation scenarios.

## 3. Results

Without vaccination, a moderate severity influenza pandemic would result in ~89 million symptomatic cases in the US, with ~718,000 hospitalizations and 206,000 deaths (Table 1). In contrast, a severe pandemic would result in ~9.6 million hospitalizations, and 2.1 million deaths. All vaccination scenarios evaluated reduced the overall estimated hospitalization and mortality burdens, with greatest reductions from use of a high effectiveness vaccine from 3 months. In line with previous analysis [7], a low effectiveness vaccine used from 3 months would lead to fewer hospitalizations and deaths overall compared with use of a high effectiveness vaccine from 6 months (Table 1).

**Figure 1:**
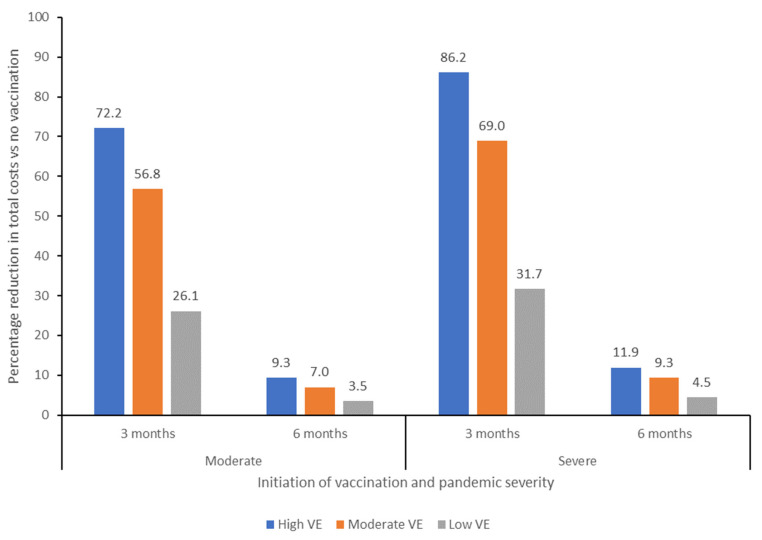
Percentage reduction in total costs compared with a no vaccination scenario of initiating vaccination at 3 or 6 months after declaration of a pandemic, for moderate and severe severity pandemic scenarios. VE, vaccine effectiveness.

Overall, the economic impact on healthcare-related costs would be greater in a late vaccination scenario compared with an early vaccination scenario in either a moderate or severe pandemic, irrespective of vaccine effectiveness. In a moderate pandemic, total costs were estimated at $116 bn in a no vaccination scenario, with greatest total cost reductions seen from vaccination initiation at 3 months and use of a high effectiveness vaccine (Table 2). Compared with the no vaccination scenario, vaccination at 3 months would result in cost reductions, excluding the cost of the vaccine itself, of 26%, 57%, and 72% for a low, moderate, and high effectiveness vaccine, respectively, corresponding to absolute reductions of $30.4 bn, $66.2 bn, and $84.1 bn, respectively. Vaccination at 6 months would result in reductions of 5%, 9%, and 12%, corresponding to absolute reductions of $4.1 bn, $8.2 bn, and $10.8 bn for the low, moderate, and high effectiveness vaccine scenarios, respectively (Figure 1). Overall, vaccination from 3 months with a low effectiveness vaccine in a moderate pandemic would result in savings of $19.6 bn compared to a high effectiveness vaccine from 6 months, excluding any differences in vaccine price.

In a severe pandemic, total costs would be higher, estimated at $823 bn in a no vaccination scenario. Initiation of vaccination at 3 months would result in a 32%, 69%, and 86% reduction in total costs (excluding vaccine cost) for a low, moderate, and high effectiveness vaccine, respectively, with absolute total cost reductions of $260.5 bn, $567.6 bn and $709.1 bn, respectively (Figure 1; Table 2. Initiation of vaccination at 6 months would reduce costs by 5–12% across all 3 vaccine scenarios, with total cost reductions of $36.7 bn, $76.8 bn, and $98.0 bn for the low, moderate, and high effectiveness vaccine scenarios, respectively. Overall, use of a low effectiveness vaccine from 3 months in a severe pandemic would result in savings of $162.6 bn compared with use of a high effectiveness vaccine from 6 months, without taking into account potential differences in vaccine price.

### Costs per Age Group

**Figure 2:**
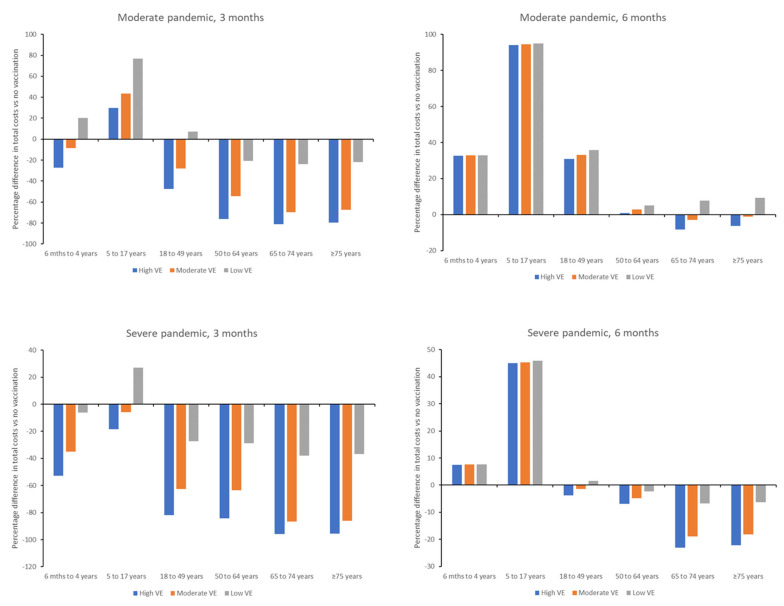
Percentage difference in total costs versus no vaccination for individual age groups for initiation of vaccination at 3 and 6 months after declaration of a moderate or severe pandemic. VE, vaccine effectiveness.

Total costs associated with each vaccination scenario were variable across age groups, with the largest percentage reduction in costs generally being observed in older age groups (Figure 2; Tables S4–S7). In the case of a moderate pandemic, vaccination at 3 months would result in cost savings versus no vaccination in adults ≥18 years of age, irrespective of vaccine effectiveness, although this may be impacted when vaccine cost is considered. Savings would also be observed with a high or moderate effectiveness vaccine in children 6 months to 4 years of age, whereas vaccination of 5–17 year-olds would be more expensive than no vaccination. Similar findings were seen in a severe pandemic, although vaccination of children <18 years of age would also be cost-saving versus no vaccination, except in the scenario of a low effectiveness vaccine in children aged 5–17 years.

Vaccination at 6 months in a moderate pandemic would only result in reduced costs versus no vaccination in the scenario of a high or moderate effectiveness vaccine, and then savings were only observed for adults ≥ 65 years (Figure 2). In a severe pandemic, costs would be reduced versus no vaccination for adults ≥ 50 years in all vaccine effectiveness scenarios, and for adults ≥ 18 years if a high or moderate effectiveness vaccine was used. Costs would be increased versus no vaccination for children 6 months to <18 years of age in both a moderate or severe pandemic.

## 4. Discussion

Our analysis demonstrates that speed of initiation of vaccination is critical in reducing the economic impact of an influenza pandemic. Mass vaccination using a high or moderate effectiveness vaccine from 3 months after declaration of a pandemic would result in cost reductions compared with no vaccination of 57–72% in a moderate pandemic and 69–86% in a severe pandemic, with reductions of 26–32% from use of a low effectiveness vaccine, excluding the price of the vaccine itself. In contrast, initiation of vaccination at 6 months after pandemic declaration would result in cost savings of 4–9% in a moderate pandemic and 5–12% in a severe pandemic, across the range of vaccine effectiveness evaluated.

The results of this analysis complement a previous evaluation of the impacts of speed of initiating vaccine roll-out on disease outcomes and healthcare system utilization [7]. In the study, we estimated that vaccination 3 months into the pandemic would prevent up to 95% of deaths compared with no vaccination, compared with only up to 21% of deaths from vaccination initiation at 6 months. While moderate and high effectiveness vaccines could flatten the pandemic curve if administered from 3 months, none of the considered vaccine effectiveness levels could impact the pandemic curve when administered from 6 months into the pandemic. Therefore, it is not surprising that our estimates of the economic impacts of delaying initiation of vaccination to 6 months are substantially higher than those for vaccination from 3 months.

We acknowledge that a major limitation of the current analysis is that vaccine prices were not available for inclusion in the model. Vaccine price during an influenza pandemic will depend on multiple factors, including when the next pandemic occurs; available vaccine platforms and technologies; availability and costs of components, raw materials, and manufacturing facilities; research and development costs; market and economic factors; quality assurance/compliance costs; and other factors [27]. Therefore, it was not justifiable to estimate a vaccine price for use in this analysis. Additionally, it would be expected that the first available vaccines (developed from related library strains) would be of lower effectiveness than specifically designed vaccines, thereby echoing the analysis performed comparing a low effectiveness vaccine (e.g., generic early vaccine) administered from 3 months with a high effectiveness vaccine (e.g., specifically designed vaccine) administered from 6 months. Although the vaccine prices were not available for inclusion in the analysis, the conclusion that speed of initiating a vaccination campaign is more impactful on minimizing costs than later vaccination with a higher effectiveness vaccine would still be valid across the potential range of vaccine prices relevant to a future influenza pandemic.

Previous analysis of potential influenza pandemic vaccination strategies in Europe (e.g., blanket vaccination, vaccination of older adults only, and vaccination of 5–19 year-olds) have been shown to be highly cost-effective [28], and studies based on the 2009 pandemic in the US have shown that earlier vaccination is more cost-saving than later vaccination [25,29]. In a simulation model based on a community of 30,000 people in Western Australia, pre-emptive vaccination with a low effectiveness vaccine, in this case prior to pandemic emergence, was found to be more cost-effective than reactive vaccination strategies [30]. Similarly, modelling studies of a potential influenza epidemic based on a US metropolitan city also demonstrated significant cost-savings associated with earlier vaccination, with higher savings the faster vaccination takes place [29,31]. Other modelling studies have also noted that antivirals and social distancing measures also play an important role in reducing the impact of an influenza pandemic, as vaccination without either of these additional interventions have been shown to be less cost-effective [3]. In our study, we assumed that 25% of symptomatic patients would also receive antivirals. While we did not consider the impact of social distancing (e.g., lockdowns), the previous epidemiology study demonstrated that this would have limited benefits without vaccination, shifting the pandemic curve temporally rather than having any substantial impact on health outcomes. While we assumed in the current analysis that mass vaccination could be initiated within 3 months of declaration of a pandemic, manufacturing, logistical or regulatory constraints may not make this achievable, in which case use of lockdowns and other social distancing measures may be vital for reducing transmission until vaccination is available.

Our analysis by age group demonstrated that economic benefits vary by age, with highest benefits seen in older adults, where savings were achieved from both reduced healthcare costs and lower mortality rates. While costs were proportionally highest for the 5–17 year age group, schoolchildren are the highest transmitters of influenza virus, therefore while vaccination of this age group may not have direct or indirect cost benefits for the vaccinated individual, the community-level benefits are likely to be substantial from reduced viral transmission [32,33]. Evaluation of which age groups would benefit from priority vaccination during a pandemic was out of scope of the current model, but may be similar to that seen during the 2009 influenza pandemic [34].

The COVID-19 pandemic has highlighted the potential economic costs and future savings associated with mass vaccination of the population. While total costs associated with the pandemic from lost GDP, premature deaths, and health impairments are estimated to be around $16 tn [2], vaccination has had a huge impact on reducing healthcare utilization and mortality rates, resulting in estimates of cost savings of $155 bn worldwide from the first 9 months of vaccination alone [35]. A US-based model also estimated that vaccination of the population would result in $1.8 to $9.9 tn gains in societal economic value across scenarios, with value derived from a combination of life years gained, healthcare costs avoided, reduced mental health issues, and gains to GDP [36]. The economic impact of a future influenza pandemic on the US will ultimately depend on the virulence/infectiousness of the pandemic virus, any pre-existing immunity within the population, which age groups are most affected, and the timing and speed of global spread. While we tried to account for this by evaluating “moderate” and “severe” pandemic scenarios, a pandemic which causes high morbidity and mortality rates in young working adults, such as seen in the 1918 pandemic, would have substantially greater (long-term) economic impacts than a virus which predominantly has serious outcomes in older adults, such as in the COVID-19 pandemic. In the current model, we assumed that those with high-risk comorbidities or age ≥ 65 years would be prioritized for vaccination; however, this may not be the case in a real influenza pandemic which, for example, has the highest rates of serious disease and mortality in children or young adults. Therefore, an effective vaccination prioritization strategy, together with ample preparedness planning could result in substantially greater economic savings than illustrated by this modelling exercise, depending on the individual dynamics of the pandemic.

As with all modelling studies, our analysis is limited by the input parameters and structure of the model. As mentioned above, we chose not to include vaccine price in the model as we did not have an accurate estimate available for pandemic influenza vaccines of differing effectiveness. As discussed earlier, we acknowledge that the vaccine price would add a substantial cost to all the vaccine scenarios; however, even with different prices for different effectiveness vaccines, the conclusion that initiation of vaccination at 3 months would result in lower economic impacts than initiation at 6 months is still relevant, as it is likely a higher effectiveness vaccine administered from 6 months would be more expensive than a low effectiveness vaccine available earlier in the pandemic. Other limitations to the model include assumptions around the transmission patterns and viral spread, underlying immunity levels, the use of a one-dose vaccine schedule, and consideration of only one vaccine formulation across age groups [7]. While not modelled in the current analysis, increased transmissibility of the pandemic virus would further increase the importance of rapid vaccination in reducing the economic and health burden of the influenza pandemic, with the potential for use of lockdowns and other social distancing measures to reduce virus spread until a vaccine becomes available. Finally, we did not consider the potential additional benefits of influenza vaccination on comorbidities and other complications, including reducing the risk of influenza-related cardiovascular events [37]. These secondary benefits would likely further reduce the economic burden of pandemic influenza, although evidence of the impacts of pandemic vaccines on cardiovascular risk remains limited.

## 5. Conclusions

In summary, our analysis has shown the speed of vaccination in an influenza pandemic has impacts on economic costs, with earlier initiation of vaccination even with a lower effectiveness vaccine resulting in overall lower direct and indirect costs compared with later vaccination with a higher effectiveness vaccine. To accelerate speed of vaccination and reduce health and economic impact, preparedness strategies could take a holistic approach potentially including stockpiling of vaccines and antivirals for rapid dissemination, as well as prioritizing steps to enable and scale more widespread vaccine roll-out to at-risk populations.

## Figures and Tables

**Table 1:** Summary of predicted health outcomes with each vaccination scenario in a moderate or severe pandemic with vaccination initiated at 3 or 6 months.

Pandemic Severity	Vaccination Scenario	Symptomatic Cases	Hospital Visits	GP Visits	Deaths
**Vaccination at 3 Months**					
Moderate	No vaccination (antiviral only)	88,942,473	717,554	39,607,982	206,487
	High effectiveness vaccine	17,940,821	91,206	6,935,595	9634
	Moderate effectiveness vaccine	36,025,035	204,249	14,776,717	32,231
	Low effectiveness vaccine	66,741,092	490,893	28,380,923	129,199
Severe	No vaccination (antiviral only)	88,942,473	9,618,426	47,529,578	2,088,333
	High effectiveness vaccine	18,626,689	1,074,635	8,892,181	90,141
	Moderate effectiveness vaccine	37,555,149	2,560,741	18,994,985	314,237
	Low effectiveness vaccine	68,419,956	6,435,691	35,434,289	1,297,349
**Vaccination at 6 Months**					
Moderate	No vaccination (antiviral only)	88,942,473	717,554	39,607,982	206,487
	High effectiveness vaccine	83,066,866	618,770	35,940,361	161,574
	Moderate effectiveness vaccine	85,006,471	637,636	37,123,270	169,058
	Low effectiveness vaccine	86,739,568	681,795	38,197,193	190,798
Severe	No vaccination (antiviral only)	88,942,473	9,618,426	47,529,578	2,088,333
	High effectiveness vaccine	83,457,744	8,226,533	43,467,459	1,627,136
	Moderate effectiveness vaccine	85,411,755	8,494,008	44,901,333	1,703,959
	Low effectiveness vaccine	87,024,741	9,111,149	46,084,171	1,926,968

GP, general practitioner.

**Table 2:** Economic costs associated with each vaccination scenario in a moderate pandemic. All costs are quoted in USD ($).

Scenario	GP Costs	Hospitalization Costs	Workday Cost	Administration Costs	Antiviral Costs	Cost of Lost Productivity Due to Excess Mortality	Total Costs
**Vaccination at 3 Months**							
No vaccination (antiviral only)	$20,599,453,279	$28,978,034,803	$12,604,418,645	$19,415,048,680	$1,118,113,040	$33,719,406,329	$116,434,474,776
High effectiveness vaccine	$2,405,596,950	$3,406,805,683	$2,592,480,598	$19,415,048,680	$209,306,610	$4,333,698,370	$32,362,936,891
Moderate effectiveness vaccine	$6,545,903,147	$8,463,078,017	$5,072,670,071	$19,415,048,680	$432,101,841	$10,346,938,544	$50,275,740,300
Low effectiveness vaccine	$13,535,209,281	$19,520,301,978	$9,288,304,732	$19,415,048,680	$835,576,668	$23,437,642,287	$86,032,083,626
**Vaccination at 6 Months**							
No vaccination (antiviral only)	$20,599,453,279	$28,978,034,803	$12,604,418,645	$19,415,048,680	$1,118,113,040	$33,719,406,329	$116,434,474,776
High effectiveness vaccine	$18,048,536,313	$25,454,554,885	$11,810,945,816	$19,415,048,680	$1,043,851,428	$29,820,764,952	$105,593,702,074
Moderate effectiveness vaccine	$18,832,091,464	$26,206,934,960	$12,049,890,261	$19,415,048,680	$1,068,215,875	$30,630,414,373	$108,202,595,613
Low effectiveness vaccine	$19,572,794,262	$27,648,503,767	$12,290,021,200	$19,415,048,680	$1,090,276,084	$32,292,287,457	$112,308,931,450

GP, general practitioner; NPI, non-pharmaceutical intervention.

## Data Availability

The data that support the findings of this study are available from the corresponding author upon reasonable request.

## Supplementary Materials

The following supporting information can be downloaded at: https://www.mdpi.com/article/10.3390/vaccines13080828/s1, Table S1: Parameters and assumption used in the epidemiological model; Table S2: Vaccine coverage rate assumptions by age group; Table S3: Economic costs by age and risk group; Table S4: Economic costs associated with vaccination at 3 months in a moderate pandemic by age group; Table S5: Economic costs associated with vaccination at 3 months in a severe pandemic by age group; Table S6: Economic costs associated with vaccination at 6 months in a moderate pandemic by age group; Table S7: Economic costs associated with vaccination at 6 months in a severe pandemic by age group.
