# Supplementary material for: Economic Impacts of Initiating Vaccination at 3 Months vs. 6 Months in an Influenza Pandemic in the United States
Source: Vaccines (Basel). 2025 Aug 1;13(8):828. doi: 10.3390/vaccines13080828 (PMC12389970; doi:10.3390/vaccines13080828)
Supplement: Supplementary file 1 [file vaccines-13-00828-s001.zip › vaccines-3736293-supplementary.pdf]

Supplementary Materials

Table S1. Parameters and assumption used in the epidemiological model (for full details see [7])

Parameter	Estimates	Reference
VE against cases		
High VE vaccine	92%	[8,9]
Moderate VE vaccine	46–88%	[10]
Low VE vaccine	24–43%	[11]
VE against hospitalization		
High VE vaccine	97%	[12]
Moderate VE vaccine	64–88%	[10]
Low VE vaccine	24–43%	[11]
Vaccine coverage	59–93%	[13,14]
Vaccination campaign duration	2 weeks with ramp up exponential function	
Antiviral effectiveness		
TTAS	-14 hours	[15,16]
Virus shedding	- 3.5 days	[17]
Influenza complications	18–70%	[18]
Mortality	18–70%	[18]
Antiviral usage	25%	[19]
Moderate pandemic		
Cases	82,650,577	[20]
Hospitalizations	10,334,945	[20]
Outpatient visits	46,250,361	[20]
Deaths	2,105,993	[20]
Severe pandemic		
Cases	82,650,577	[20]
Hospitalizations	813,243	[20]
Outpatient visits	38,541,967	[20]
Deaths	208,588	[20]
Pandemic duration	180 days	[21]
Duration of hospital stay – non-ICU	5 days	[22,23]
Duration of hospital stay - ICU	7 days	[22,23]

VE, vaccine effectiveness; TTAS, time to alleviate symptoms

Table S2. Vaccine coverage rate assumptions by age group [13,14]

**Age**	**Coverage rate**
6 months – 4 years	68%
5 – 17 years	59%
18 – 49 years	74%
50 – 64 years	74%
65 – 74 years	93%
≥75 years	93%

Table S3. Economic costs by age and risk group [24,25]. All costs are quoted in USD ($)

Age group	GP cost per consultation ($)		Hospital cost per episode ($)		QALY loss per nonfatal ILI case	QALY loss per hospitalisation	Lost workday - cases	Lost workday - outpatient visit		Lost workday - hospitalization		Workday costs ($)
	LR	HR	LR	HR				LR	HR	LR	HR	
6 months-4 years	57	57	8,513	13,395	0.005	0.076	1	1	6	8	31	103
5-17 years	57	57	9,608	12,913	0.005	0.076	0.5	1	4	9	23	103
18-49 years	195	1135	29,770	74,709	0.007	0.013	0.5	1	2	12	21	103
50-64 years	235	1147	34,926	64,670	0.007	0.013	0.5	2	4	13	24	106
65-74 years	378	746	17,931	26,223	0.007	0.013	1	3	7	13	18	15
≥75 years	378	746	17,931	26,223	0.007	0.013	1	3	7	13	18	15

GP, general practitioner; HR, high risk; ILI, influenza-like illness; LR, low risk; QALY, quality-adjusted life years

Table S4. Economic costs associated with vaccination at 3 months in a moderate pandemic by age group. All costs are quoted in USD ($)

Scenario	GP Costs	Hospitalization Costs	Workday Cost	Administration Costs	Antiviral Costs	Cost of lost productivity due to excess mortality	Total Costs
**6 months to 4 years old**							
No vaccination (antiviral only)	$205,070,982	$757,311,611	$1,408,632,870	$0	$93,347,766	$442,602,873	$2,906,966,100
High effectiveness vaccine	$82,489,936	$298,040,262	$562,954,888	$962,072,541	$35,596,058	$173,574,530	$2,114,728,215
Moderate effectiveness vaccine	$122,502,714	$447,529,653	$811,253,944	$962,072,541	$55,315,002	$259,412,240	$2,658,086,095
Low effectiveness vaccine	$180,398,638	$663,889,937	$1,212,826,331	$962,072,541	$82,817,186	$386,173,872	$3,488,178,504
**5 to 17 years old**							
No vaccination (antiviral only)	$292,622,720	$82,212,152	$1,821,798,992	$0	$215,183,347	$231,934,304	$2,643,751,515
High effectiveness vaccine	$98,502,272	$28,105,086	$632,505,858	$2,523,428,442	$65,875,127	$80,668,335	$3,429,085,121
Moderate effectiveness vaccine	$142,968,307	$39,814,871	$876,936,264	$2,523,428,442	$102,726,348	$111,356,951	$3,797,231,183
Low effectiveness vaccine	$240,739,316	$67,273,808	$1,477,704,885	$2,523,428,442	$178,923,582	$188,289,253	$4,676,359,286
**18 to 49 years old**							
No vaccination (antiviral only)	$7,059,463,684	$6,947,908,607	$3,293,433,360	$0	$344,672,640	$3,248,359,710	$20,893,838,000
High effectiveness vaccine	$933,926,872	$856,250,956	$477,477,982	$8,182,412,182	$48,489,991	$442,833,216	$10,941,391,199
Moderate effectiveness vaccine	$2,347,512,837	$2,189,297,085	$1,148,844,203	$8,182,412,182	$117,422,271	$1,084,243,844	$15,069,732,423
Low effectiveness vaccine	$4,699,754,105	$4,648,640,707	$2,315,250,741	$8,182,412,182	$248,769,146	$2,271,717,944	$22,366,544,826
**50 to 64 years old**							

No vaccination (antiviral only)	$7,223,908,960	$14,250,552,431	$5,029,791,490	$0	$290,939,469	$20,431,104,063	$47,226,296,413
High effectiveness vaccine	$1,136,229,834	$2,069,594,961	$891,967,189	$3,725,200,730	$55,219,281	$3,428,855,159	$11,307,067,154
Moderate effectiveness vaccine	$2,709,410,674	$5,017,321,529	$2,021,792,189	$3,725,200,730	$122,728,303	$7,856,134,322	$21,452,587,746
Low effectiveness vaccine	$5,011,916,118	$9,944,864,721	$3,666,662,081	$3,725,200,730	$223,842,817	$14,934,961,049	$37,507,447,516
**65 to 74 years old**							
No vaccination (antiviral only)	$3,327,613,908	$3,937,722,170	$600,568,776	$0	$99,472,509	$6,057,123,301	$14,022,500,664
High effectiveness vaccine	$85,680,777	$85,183,569	$15,287,268	$2,342,367,415	$2,282,445	$131,074,819	$2,661,876,294
Moderate effectiveness vaccine	$691,827,812	$431,460,621	$120,871,581	$2,342,367,415	$19,147,249	$663,869,342	$4,269,544,020
Low effectiveness vaccine	$1,935,997,174	$2,367,935,845	$350,193,034	$2,342,367,415	$57,568,177	$3,642,910,926	$10,696,972,571
**≥75 years old**							
No vaccination (antiviral only)	$2,490,773,025	$3,002,327,833	$450,193,156	$0	$74,497,310	$3,308,282,079	$9,326,073,402
High effectiveness vaccine	$68,767,258	$69,630,848	$12,287,414	$1,679,567,352	$1,843,709	$76,692,311	$1,908,788,891
Moderate effectiveness vaccine	$531,680,804	$337,654,258	$92,971,890	$1,679,567,352	$14,762,669	$371,921,843	$3,028,558,816
Low effectiveness vaccine	$1,466,403,931	$1,827,696,961	$265,667,660	$1,679,567,352	$43,655,759	$2,013,589,244	$7,296,580,907

GP, general practitioner

Table S5. Economic costs associated with vaccination at 3 months in a severe pandemic by age group. All costs are quoted in USD ($)

Scenario	GP Costs	Hospitalization Costs	Workday Cost	Administration Costs	Antiviral Costs	Cost of lost productivity due to excess mortality	Total Costs
**6 months to 4 years old**							
No vaccination (antiviral only)	$246,085,178	$6,754,811,287	$2,320,591,207	$0	$99,722,167	$2,917,078,497	$12,338,288,336
High effectiveness vaccine	$104,094,640	$2,617,785,107	$966,552,297	$962,072,541	$39,323,317	$1,124,436,863	$5,814,264,764
Moderate effectiveness vaccine	$152,045,355	$3,849,728,263	$1,350,775,521	$962,072,541	$60,231,612	$1,641,308,771	$8,016,162,062
Low effectiveness vaccine	$219,523,447	$5,800,238,001	$1,994,551,295	$962,072,541	$89,135,366	$2,486,730,232	$11,552,250,882
**5 to 17 years old**							
No vaccination (antiviral only)	$351,147,264	$876,178,376	$2,121,610,960	$0	$215,893,422	$1,882,699,578	$5,447,529,601
High effectiveness vaccine	$118,511,685	$311,781,836	$745,416,329	$2,523,428,442	$66,242,257	$681,372,308	$4,446,752,857
Moderate effectiveness vaccine	$171,872,365	$415,733,905	$1,019,511,741	$2,523,428,442	$103,184,017	$885,297,405	$5,119,027,874
Low effectiveness vaccine	$289,230,850	$703,635,008	$1,717,139,314	$2,523,428,442	$179,627,911	$1,499,504,335	$6,912,565,861
**18 to 49 years old**							
No vaccination (antiviral only)	$8,471,356,421	$105,640,612,578	$6,507,637,871	$0	$358,404,138	$30,268,655,825	$151,246,666,833
High effectiveness vaccine	$1,207,223,928	$12,897,404,045	$898,135,021	$8,182,412,182	$51,530,613	$3,942,307,185	$27,179,012,974
Moderate effectiveness vaccine	$3,024,301,113	$33,113,942,362	$2,218,242,163	$8,182,412,182	$124,945,725	$9,840,376,313	$56,504,219,859

Low effectiveness vaccine	$5,881,981,231	$70,399,716,243	$4,525,952,390	$8,182,412,182	$261,628,032	$20,742,366,386	$109,994,056,466
**50 to 64 years old**							
No vaccination (antiviral only)	$8,668,690,752	$212,774,334,709	$13,513,312,700	$0	$322,782,988	$196,100,103,876	$431,379,225,025
High effectiveness vaccine	$1,597,237,593	$29,944,109,651	$2,205,696,856	$3,725,200,730	$63,398,005	$30,327,844,732	$67,863,487,567
Moderate effectiveness vaccine	$3,787,589,531	$73,539,635,369	$5,216,435,251	$3,725,200,730	$142,056,305	$71,696,331,078	$158,107,248,263
Low effectiveness vaccine	$6,596,746,404	$147,083,165,894	$9,798,229,185	$3,725,200,730	$254,728,999	$139,560,413,903	$307,018,485,114
**65 to 74 years old**							
No vaccination (antiviral only)	$3,993,136,690	$56,488,746,166	$1,227,096,070	$0	$120,313,826	$62,009,292,698	$123,838,585,450
High effectiveness vaccine	$178,688,465	$1,220,799,042	$41,341,119	$2,342,367,415	$4,031,848	$1,340,369,532	$5,127,597,421
Moderate effectiveness vaccine	$1,037,701,196	$6,184,375,503	$231,415,761	$2,342,367,415	$25,168,560	$6,789,893,679	$16,610,922,114
Low effectiveness vaccine	$2,563,985,723	$33,955,812,644	$763,629,368	$2,342,367,415	$74,582,903	$37,277,212,085	$76,977,590,138
**≥75 years old**							
No vaccination (antiviral only)	$2,988,927,630	$41,673,993,262	$913,133,971	$0	$90,069,022	$33,823,589,081	$79,489,712,965
High effectiveness vaccine	$141,929,486	$965,212,109	$32,829,232	$1,679,567,352	$3,255,403	$783,170,452	$3,605,964,035
Moderate effectiveness vaccine	$792,577,034	$4,681,464,712	$176,469,535	$1,679,567,352	$19,387,545	$3,798,687,514	$11,148,153,693

Low effectiveness vaccine	$1,936,293,902	$25,355,772,292	$574,332,212	$1,679,567,352	$56,524,306	$20,577,045,908	$50,179,535,972

GP, general practitioner

Table S6. Economic costs associated with vaccination at 6 months in a moderate pandemic by age group. All costs are quoted in USD ($)

Scenario	GP Costs	Hospitalization Costs	Workday Cost	Administration Costs	Antiviral Costs	Cost of lost productivity due to excess mortality	Total Costs
**6 months to 4 years old**							
No vaccination (antiviral only)	$205,070,982	$757,311,611	$1,408,632,870	$0	$93,347,766	$442,602,873	$2,906,966,100
High effectiveness vaccine	$204,151,402	$754,048,863	$1,403,015,209	$962,072,541	$92,906,968	$440,746,181	$3,856,941,165
Moderate effectiveness vaccine	$204,551,147	$755,464,946	$1,405,436,933	$962,072,541	$93,098,725	$441,550,663	$3,862,174,955
Low effectiveness vaccine	$204,836,799	$756,479,886	$1,407,191,346	$962,072,541	$93,235,488	$442,128,885	$3,865,944,944
**5 to 17 years old**							
No vaccination (antiviral only)	$292,622,720	$82,212,152	$1,821,798,992	$0	$215,183,347	$231,934,304	$2,643,751,515
High effectiveness vaccine	$288,735,307	$81,105,169	$1,797,659,759	$2,523,428,442	$212,316,097	$228,806,765	$5,132,051,540
Moderate effectiveness vaccine	$289,991,089	$81,434,223	$1,804,376,043	$2,523,428,442	$213,362,575	$229,659,484	$5,142,251,857
Low effectiveness vaccine	$291,419,114	$81,861,034	$1,813,770,419	$2,523,428,442	$214,357,078	$230,906,232	$5,155,742,319
**18 to 49 years old**							
No vaccination (antiviral only)	$7,059,463,684	$6,947,908,607	$3,293,433,360	$0	$344,672,640	$3,248,359,710	$20,893,838,000
High effectiveness vaccine	$6,460,519,200	$6,290,568,130	$3,086,456,400	$8,182,412,182	$327,931,079	$3,009,358,378	$27,357,245,369
Moderate effectiveness vaccine	$6,622,337,685	$6,459,318,227	$3,147,711,492	$8,182,412,182	$333,385,204	$3,075,478,656	$27,820,643,447

Low effectiveness vaccine	$6,796,285,751	$6,675,211,595	$3,209,326,405	$8,182,412,182	$338,384,777	$3,153,518,595	$28,355,139,305
**50 to 64 years old**							
No vaccination (antiviral only)	$7,223,908,960	$14,250,552,431	$5,029,791,490	$0	$290,939,469	$20,431,104,063	$47,226,296,413
High effectiveness vaccine	$6,692,539,972	$13,112,318,212	$4,729,075,842	$3,725,200,730	$278,960,337	$19,107,369,800	$47,645,464,893
Moderate effectiveness vaccine	$6,840,490,560	$13,416,756,434	$4,815,313,153	$3,725,200,730	$282,679,193	$19,473,681,013	$48,554,121,083
Low effectiveness vaccine	$6,994,893,521	$13,793,150,098	$4,904,840,639	$3,725,200,730	$286,212,802	$19,909,811,057	$49,614,108,847
**65 to 74 years old**							
No vaccination (antiviral only)	$3,327,613,908	$3,937,722,170	$600,568,776	$0	$99,472,509	$6,057,123,301	$14,022,500,664
High effectiveness vaccine	$2,508,233,684	$2,948,029,876	$452,488,440	$2,342,367,415	$75,032,387	$4,535,060,526	$12,861,212,327
Moderate effectiveness vaccine	$2,780,906,249	$3,108,119,041	$500,024,333	$2,342,367,415	$83,090,640	$4,781,279,067	$13,595,786,745
Low effectiveness vaccine	$3,018,557,850	$3,593,224,875	$545,009,821	$2,342,367,415	$90,261,903	$5,527,376,538	$15,116,798,402
**≥75 years old**							
No vaccination (antiviral only)	$2,490,773,025	$3,002,327,833	$450,193,156	$0	$74,497,310	$3,308,282,079	$9,326,073,402
High effectiveness vaccine	$1,894,356,749	$2,268,484,635	$342,250,165	$1,679,567,352	$56,704,561	$2,499,423,301	$8,740,786,763
Moderate effectiveness vaccine	$2,093,814,734	$2,385,842,089	$377,028,307	$1,679,567,352	$62,599,538	$2,628,765,489	$9,227,617,509

Low effectiveness vaccine	$2,266,801,227	$2,748,576,279	$409,882,570	$1,679,567,352	$67,824,035	$3,028,546,151	$10,201,197,615

GP, general practitioner

Table S7. Economic costs associated with vaccination at 6 months in a severe pandemic by age group. All costs are quoted in USD ($)

Scenario	GP Costs	Hospitalization Costs	Workday Cost	Administration Costs	Antiviral Costs	Cost of lost productivity due to excess mortality	Total Costs
**6 months to 4 years old**							
No vaccination (antiviral only)	$246,085,178	$6,754,811,287	$2,320,591,207	$0	$99,722,167	$2,917,078,497	$12,338,288,336
High effectiveness vaccine	$244,984,190	$6,729,036,605	$2,312,414,589	$962,072,541	$99,255,732	$2,906,444,505	$13,254,208,162
Moderate effectiveness vaccine	$245,463,769	$6,740,133,633	$2,315,922,297	$962,072,541	$99,458,749	$2,911,007,758	$13,274,058,748
Low effectiveness vaccine	$245,805,338	$6,748,195,244	$2,318,485,079	$962,072,541	$99,603,413	$2,914,341,180	$13,288,502,795
**5 to 17 years old**							
No vaccination (antiviral only)	$351,147,264	$876,178,376	$2,121,610,960	$0	$215,893,422	$1,882,699,578	$5,447,529,601
High effectiveness vaccine	$346,496,346	$864,340,224	$2,093,597,905	$2,523,428,442	$213,021,482	$1,857,224,466	$7,898,108,866
Moderate effectiveness vaccine	$348,000,668	$867,175,600	$2,101,050,688	$2,523,428,442	$214,069,527	$1,862,690,662	$7,916,415,588
Low effectiveness vaccine	$349,710,506	$872,103,133	$2,112,154,781	$2,523,428,442	$215,066,593	$1,873,632,065	$7,946,095,521
**18 to 49 years old**							
No vaccination (antiviral only)	$8,471,356,421	$105,640,612,578	$6,507,637,871	$0	$358,404,138	$30,268,655,825	$151,246,666,833
High effectiveness vaccine	$7,794,403,789	$95,450,519,494	$6,010,993,877	$8,182,412,182	$340,987,111	$27,745,750,802	$145,525,067,255
Moderate effectiveness vaccine	$7,988,521,013	$98,052,853,798	$6,149,606,749	$8,182,412,182	$346,772,839	$28,417,188,349	$149,137,354,930

Low effectiveness vaccine	$8,186,915,475	$101,400,938,616	$6,306,338,771	$8,182,412,182	$352,044,195	$29,243,521,305	$153,672,170,545
**50 to 64 years old**							
No vaccination (antiviral only)	$8,668,690,752	$212,774,334,709	$13,513,312,700	$0	$322,782,988	$196,100,103,876	$431,379,225,025
High effectiveness vaccine	$8,123,119,191	$195,140,674,624	$12,560,348,320	$3,725,200,730	$309,578,231	$181,670,803,860	$401,529,724,957
Moderate effectiveness vaccine	$8,298,143,745	$199,831,659,674	$12,830,332,826	$3,725,200,730	$313,947,440	$185,579,220,709	$410,578,505,124
Low effectiveness vaccine	$8,459,583,190	$205,666,122,244	$13,136,919,001	$3,725,200,730	$317,983,139	$190,344,304,998	$421,650,113,301
**65 to 74 years old**							
No vaccination (antiviral only)	$3,993,136,690	$56,488,746,166	$1,227,096,070	$0	$120,313,826	$62,009,292,698	$123,838,585,450
High effectiveness vaccine	$3,100,569,671	$42,282,312,267	$936,427,995	$2,342,367,415	$92,281,764	$46,416,408,304	$95,170,367,415
Moderate effectiveness vaccine	$3,434,981,804	$44,579,874,077	$1,014,264,198	$2,342,367,415	$101,320,211	$48,938,288,588	$100,411,096,293
Low effectiveness vaccine	$3,689,158,290	$51,541,985,776	$1,126,905,427	$2,342,367,415	$110,498,721	$56,580,136,737	$115,391,052,366
**≥75 years old**							
No vaccination (antiviral only)	$2,988,927,630	$41,673,993,262	$913,133,971	$0	$90,069,022	$33,823,589,081	$79,489,712,965
High effectiveness vaccine	$2,339,065,421	$31,478,998,373	$702,832,169	$1,679,567,352	$69,702,933	$25,547,611,482	$61,817,777,730
Moderate effectiveness vaccine	$2,583,028,198	$33,108,947,999	$759,179,028	$1,679,567,352	$76,294,347	$26,870,678,004	$65,077,694,928

Low effectiveness vaccine	$2,768,041,235	$38,147,008,065	$840,964,628	$1,679,567,352	$82,979,419	$30,960,205,935	$74,478,766,634

GP, general practitioner
